# Complex association of self-rated health, depression, functional ability with loneliness in rural community-dwelling older people

**DOI:** 10.1186/s12877-023-03965-4

**Published:** 2023-05-04

**Authors:** Wenwen Cao, Chenglin Cao, Bohua Ren, Jing Yang, Ruoling Chen, Zhi Hu, Zhongliang Bai

**Affiliations:** 1 Department of Health Services Management, School of Health Services Management, Anhui Medical University, Hefei, 230032 China; 2 Department of Epidemiology and Biostatistics, School of Public Health, Guangdong Medical University, Dongguan, 523808 China; 3 Faculty of Education, Health and Wellbeing, University of Wolverhampton, Wolverhampton, WV1 1QU UK; 4 Educational Institute of behavioral medicine, Jining Medical University, Jining, 272067 China

**Keywords:** Loneliness, Self-rated health, Older people, Cross-sectional, China

## Abstract

**Background:**

This study aimed to explore whether and how self-rated health, depression and functional ability interactively associated with loneliness using a sample group of older adults residing in China rural communities.

**Methods:**

Data on socio-demographic information, self-rated health, depressive symptoms, functional ability and loneliness (quantified via a single question) were collected from 1009 participants. Cross-tabulations with chi-square test, bivariate correlations, and Classification and Regression Tree (CART) models were employed for analysis.

**Results:**

We found that 45.1% of the participants were classified as lonely. Our results gain insight into the hierarchical order of predictors for the presence of loneliness, suggesting that there was a significant interaction between functional ability and depressive symptoms while self-rated health was not a significant factor. The probability of loneliness increased with the combination of limited functional ability and depression, and varied with different interaction of functional ability, depressive symptoms, and marital status, respectively. Notably, while there were some differences, similar associations were observed among older male and female respondents.

**Conclusion:**

To delay or reduce loneliness, early detection which focuses on older people who report limitations in functional ability, depression, and being female, offers opportunities to start early interventions. Our findings might be helpful not only in designing and implementing loneliness prevention programs but also in improving healthcare for older rural community-dwelling people.

## Background

The aging population is growing rapidly, and there is consequently a greater focus on strategies to improve their health and well-being worldwide [1]. Loneliness, defined as a subjective perception of social isolation and negative feeling, has been shown to adversely impact physical and psychological health [2], resulting in a lower quality of life among older people [1]. Loneliness is regarded as a severe public health issue. Therefore, identifying the risk factors and developing intervention programs for loneliness in later life is of great importance [3]. Evidence suggests that factors such as retirement, living alone, loss of family members, physical inactivity and a lack of social capital are all associated with loneliness [4].

Researchers have documented other factors that are also associated with loneliness. For instance, a study found that poor self-rated health was a risk factor [5] and other studies reported that older people living in rural areas with poor health conditions were more likely to experience loneliness [3]. Previous studies have also revealed that being entirely or partly dependent on performing activities of daily living (ADL) and instrumental activities of daily living (IADL) were factors that contributed to one’s level of loneliness [6, 7]. In addition, some research concluded that mental health issues like depressive symptoms could trigger feelings of loneliness [8].

The above-mentioned findings are very relevant in preventing feelings of loneliness and alleviating the health issues of older people. Although these studies examined the factors independently associated with loneliness, they did not explore how they interacted to have negative impacts or whether these connections had a combined effect on loneliness. In practice, the existence of additional factors could also influence loneliness. A study from Finland revealed that one’s living status and self-rated health had a synergetic effect on loneliness, suggesting that older people with poor self-rated health and who lived on their own were most likely to experience a sense of loneliness. Living status and self-rated health emerged as the most significant explanatory factors for experiencing loneliness [9]. Exploring the combined effects of all these factors on loneliness may be necessary for subsequent relevant interventions. Furthermore, identifying the factor with the strongest association may help draft and design more targeted and focused intervention programs.

Older people are attracting increasing social attention, especially when it comes to loneliness in those living in rural communities [3, 10]. The older people in rural China are considered a vulnerable demographic, being more likely to suffer from loneliness than their urban counterparts [3, 11]. Empirical studies have shown that the prevalence of loneliness in rural older people is quite high from 25 to 78.1% [10, 11] with gender differences [12], underlining the importance of addressing loneliness issues in older adults, particularly those living in rural communities.

Therefore, this study aims to explore the interactions and combinations of self-rated health, depression, and functional ability on the presence of loneliness among older rural community-dwelling people, with a special focus on sex differences.

## Methods

### Data sources

Data analyzed in this paper was derived from our cross-sectional survey, which was conducted from July to September 2017 in Anhui Province, investigated and 24 communities and villages, and interviewed 1935 older people finally [13–15]. We conducted this study via a multi-stage stratified cluster random sampling method to obtain a representative sample age of ≥ 60 years. Data were collected through structured questionnaires face to face. This study included 1009 participants who resided in rural communities for analysis. Detailed information on our study design and data collection process has been described previously [13–15].

### Dependent variable

Herein, we took the variable of loneliness as the outcome for analysis, measured by one single question. During the interview, participants were asked whether they had feelings of loneliness and were asked to choose from three options: ‘often’, ‘sometimes’, and ‘never’. To compare with previous studies, our data analysis dichotomized loneliness into two classifications: with or without loneliness [2]. We chose one question to measure because of some advantages of using one single question to measure loneliness like brevity, refrained respondent fatigue, and popularity among older communities proven from the previous study [2]. The description of the measurement and calculation of outcome variables can be found in our previous work [14].

### Independent variables

Our main independent variables include self-rated health, ADL and IADL, and mental health depressive symptoms. Our study assessed self-rated health using one general question -“How do you rate your general health?”. Responses ranged from ‘very poor’, ‘poor’, ‘fair’, ‘good’, and ‘very good’. For data analysis, we dichotomized the original responses into a binary variable: good health (0 = ‘good’ or ‘very good’) and poor health (1 = ‘very poor’, ‘poor’, or ‘fair’), which was similar to previous studies [16, 17]. As a subjective indicator of health, self-rated health was typically measured with a single item, which was reliable and valid based on findings from previous studies [18–20].

The participants’ functional health was assessed using 14 items, including eight ADL items and six IADL items, with Cronbach’s α of this tool being 0.925, indicating good reliability in this sample. If a participant’s answer was partially limited or dependent on any item, they were classified as having “limited”, otherwise, classified as having “robust” [21]. More content concerning this measurement tool has been reported in our previous work [15]. The 16 adopted Zung Self-Rating Depression Scale (SDS) items were used to measure the participants’ depressive symptoms [22]. We summated up the items to calculate the total depression score, ranging from 16 to 64, with higher scores indicating a lower likelihood of depression. For data analysis, we dichotomized depressive symptoms into two categories, namely ‘depressed’ and ‘normal’, according to the median score [23]. More details about the measurement can be found in previous studies [13, 15].

### Other variables

Besides the above variables, socio-demographic data (e.g., age, sex, educational attainment), living arrangement, marital status, number of diseases, alcohol, cigarette smoking, and body mass index (BMI) were also collected. These variables have been elaborated in our previous studies [13–15].

### Statistical analysis

Cross-tabulations with a chi-square test were fitted to examine the differences between men and women. The association of included variables with loneliness were then examined using Kendall’s Tau-b statistic. Classification and regression tree models (CART) were next developed to assess the complex associations between functional health and loneliness. The models in men and women were also fitted separately. In the CART model, the most explanatory independent variables were used to divide loneliness (independent variables) into subgroups, with any potential interaction and combination of each independent variable generating these subgroups in the node.

In this model, loneliness (dependent variables) was categorized into subgroups by the most explanatory independent variables. Any possible interaction and combination of each independent variable could generate these subgroups in the node. Factors with statistical significance were included in the model. More information on the CART model can be found in our previous papers [13–15]. All statistical analyses were conducted using Statistical Package for the Social Sciences, version 23.0 (SPSS Inc., Chicago, IL, USA).

## Results

### Descriptive statistics

The descriptive statistics of all variables in this study are shown in Table 1. Males and females made up 45.0% and 55.0% of the participants in the study respectively. 45.1% of the participants were classified as lonely. Statistical differences between men and women were observed for multiple variables, including age, education, marital status, smoking status, drinking status, Body Mass Index, self-rated health, functional ability, and depressive symptoms except for living arrangement and the number of diseases.

For self-rated health, 23.1% of the participants reported good self-rated health, while 76.9% reported poor self-rated health. For functional ability, the percentage of “robust” and “limited” functionality was 47.1% and 52.9%, respectively. More than half of the participants reported depressive symptoms (56.4%).

**Table 1:** Demographic characteristic of the rural older people stratified by sex [N (%)]

Variables		Total (N = 1009)	Sex		
			Male 454(45.0)	Female 555(55.0)	*P*-value
**Age (years)**	60–69	447(44.3)	183(40.3)	264(47.6)	0.020
	70–79	401(39.7)	185(40.7)	216(38.9)	
	≥ 80	161(16.0)	86(18.9)	75(13.5)	
**Education**	Primary school and below	839(83.2)	328(72.2)	511(92.1)	< 0.001
	Junior school	118(11.7)	85(18.7)	33(5.9)	
	High school and above	52(5.2)	41(9.0)	11(2.0)	
**Living arrangement**	Not living alone	850(84.2)	381(83.9)	469(84.5)	0.800
	Living alone	159(15.8)	73(16.1)	86(15.5)	
**Marital status**	Married/cohabited	776(76.9)	364(80.2)	412(74.2)	0.026
	Single	233(23.1)	90(19.8)	143(25.8)	
**Smoking status**	Non-smoking	763(75.6)	223(49.1)	540(97.3)	< 0.001
	Former smoking	45(4.5)	42(9.3)	3(0.5)	
	Smoking	201(19.9)	189(41.6)	12(2.2)	
**Drinking status**	Non-drinking	817(81.00	289(63.7)	528(95.1)	< 0.001
	Former drinking	39(3.9)	34(7.5)	5(0.9)	
	Drinking	153(15.2)	131(28.9)	22(4.0)	
**Body massive index (kg/m** ^**2**^ **)**	< 18.5	135(13.4)	69(15.2)	66(11.9)	0.013
	18.5–22.9	486(48.2)	208(45.8)	278(50.1)	
	23.0-27.4	314(31.1)	154(33.9)	160(28.8)	
	≥ 27.5	74(7.3)	23(5.1)	51(9.2)	
**Number of disease**	0	291(28.8)	155(34.1)	136(24.5)	0.800
	1	408(40.4)	193(42.5)	215(38.7)	
	2	186(18.4)	73(16.1)	113(20.4)	
	> 2	124(12.3)	33(7.3)	91(16.4)	
**Self-rated health**	Poor	776(76.9)	325(71.6)	451(81.3)	< 0.001
	Good	233(23.1)	129(28.4)	104(18.7)	
**Functional ability**	Robust	475(47.1)	274(60.4)	201(36.2)	< 0.001
	Limited	534(52.9)	180(39.6)	354(63.8)	
**Depressive symptoms**	Normal	440(43.6)	219(48.2)	221(39.8)	0.007
	Depressed	569(56.4)	235(51.8)	334(60.2)	
**Loneliness**	Yes	455(45.1)	184(40.5)	271(48.8)	0.008
	No	554(54.9)	270(59.5)	284(51.2)	

### Correlation analysis

Table 2 presents the bivariate correlation of those variables with loneliness, demonstrating significant correlation, except for education, smoking, drinking, Body Mass Index (P > 0.05).

**Table 2:** Correlation of included variables and loneliness

Variables	Loneliness		
	**r**	***P*** **-value**	
Age	0.077	0.010	
Living arrangement	0.106	0.001	
Marital status	0.160	< 0.001	
Education	-0.018	0.563	
Smoking	-0.047	0.131	
Drinking	-0.056	0.071	
Body massive index	-0.053	0.073	
Self-rated health	0.142	< 0.001	
Number of disease	0.150	< 0.001	
Functional ability	0.340	< 0.001	
Depressive symptoms	0.219	< 0.001	

Note: r refers to correlations efficient

### Regression tree analysis

**Fig. 1:**
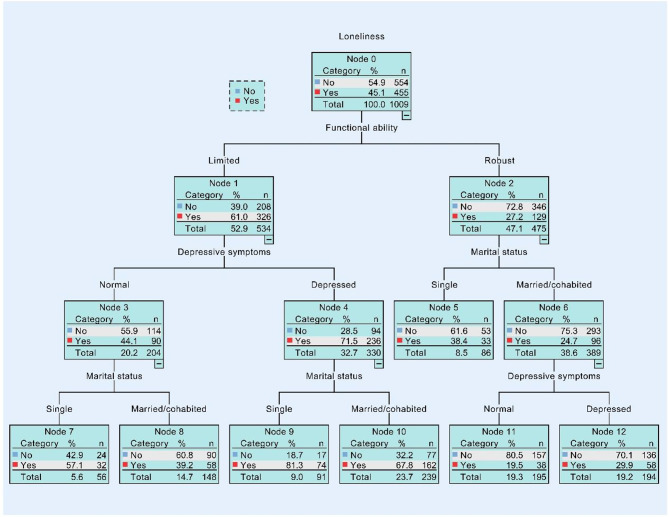
Output of CART tree model in all rural samples. (This regression tree displays factors linked to the development of loneliness and the likelihood of experiencing loneliness in the node boxes, from which the combined associations of self-rated health, depression, and functional ability can be obtained. This tree includes all rural participants.)

Fig. 1 shows the apparent cumulative associations between different variables and loneliness, indicating that different variables played different roles in the development of loneliness. Functional ability was the primary classifying factor, implying that this variable was the most significant in determining loneliness among all the respondents in our study.

For those who were limited in functional ability, the probability of loneliness could increase from 45.1 to 61.0%, while it could decline to 27.2% if the functional ability were robust. Also, the relationship between depressive symptoms and marital status in relation to loneliness was observed; for the depressed group, those being single in marital status had a higher likelihood of experiencing loneliness (81.3%) than those being married/cohabited (67.8%). Meanwhile, the mixed combinations of depressive symptoms and marital status related to loneliness were then observed. By comparison, those with a robust functional capability while married or cohabitated and who had no depressive symptoms were the least likely to experience loneliness (19.5%).

**Fig. 2:**
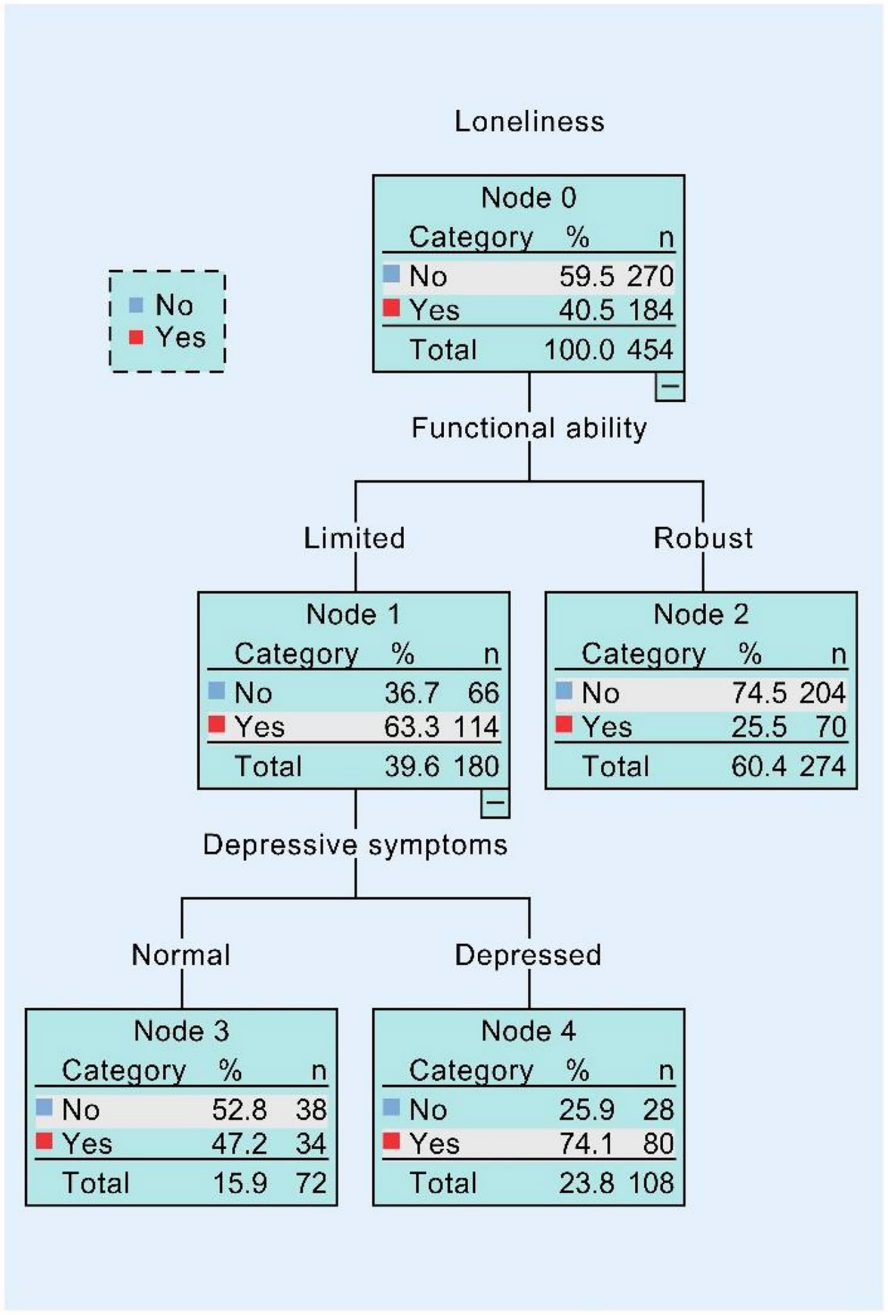
Output of CART tree model in rural male samples. (This regression tree displays factors linked to the development of loneliness and the likelihood of experiencing loneliness in the node boxes, from which the combined associations of self-rated health, depression, and functional ability can be obtained. This tree includes rural male participants.)

**Fig. 3:**
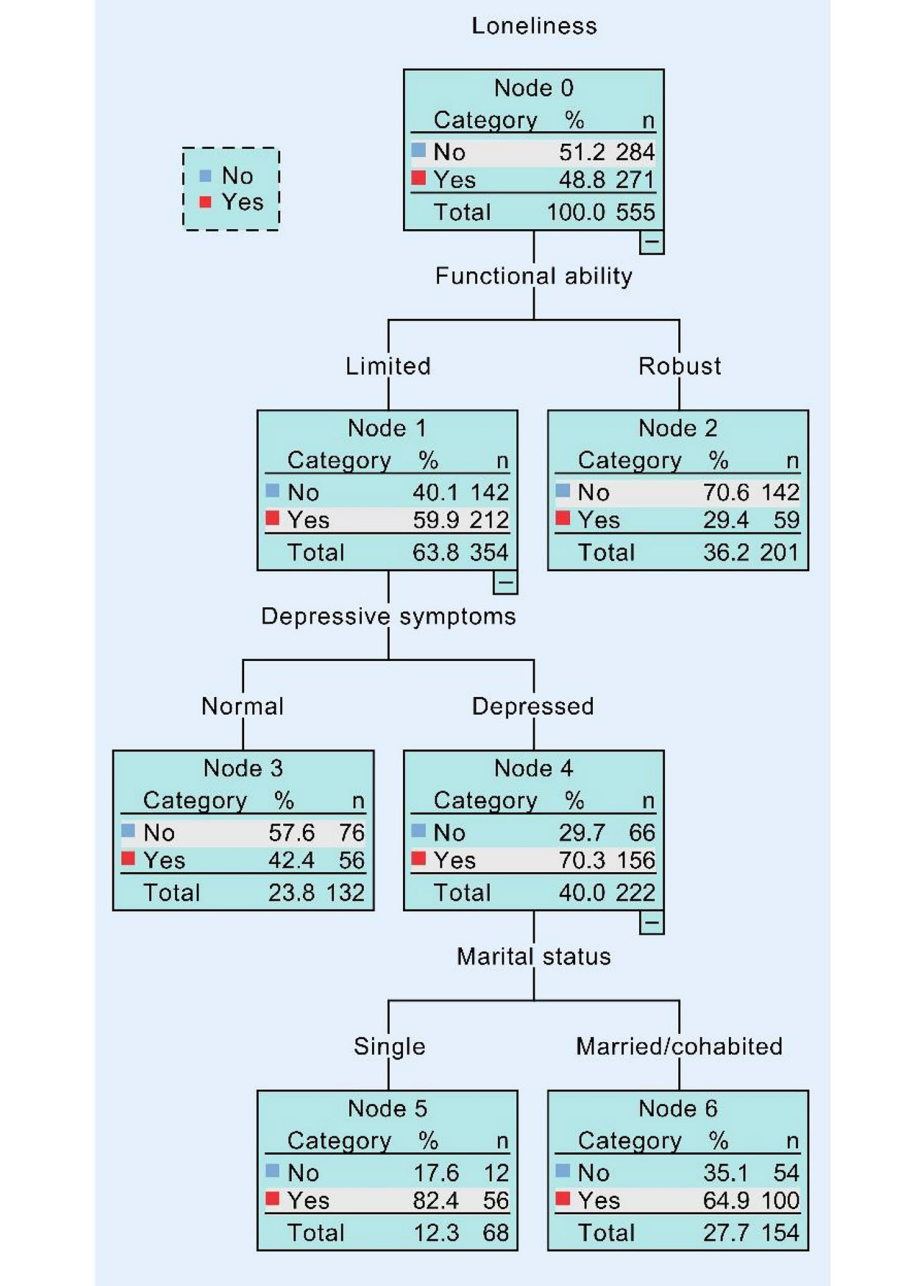
Output of CART tree model in rural female samples. (This regression tree displays factors linked to the development of loneliness and the likelihood of experiencing loneliness in the node boxes, from which the combined associations of self-rated health, depression, and functional ability can be obtained. This tree includes rural female participants.)

The above associations were also observed in older male and female respondents separately. As shown in Fig. 2, functional ability and depressive symptoms were all found to correlate with loneliness in rural male residents. 40.5% of the male participants were classified as lonely. After answering “Limited” to the question regarding functional ability, this figure amounted to 63.3%. Once the respondents answered the question about depressive symptoms positively, this figure climbed to 74.1%. On the other hand, as seen in Fig. 3, the functional ability was also a predominant factor to predict loneliness. The probability of loneliness was 59.9% for participants with limited functional ability, whereas it was 29.4% for those with robust functional ability. Depressive symptoms while being single in marital status might have a higher probability of loneliness (82.4%) than those being married/cohabited (64.9%).

## Discussion

This paper first revealed variables that could collectively identify individuals at risk of loneliness, with functional ability being the predominant determinant. The complex associations of variables observed in the stratified regression tree were identical to the findings reported for the total sample. More notably, the stratified regression tree analysis results indicated that this combination persisted differently in older males and females.

In reality, many older rural residents have become empty-nesters as a result of the younger generation’s migration to cities during China’s rapid urbanization process, which also explains why loneliness is so prevalent in rural communities [10]. We discovered that 45.1% of the older residents experienced loneliness, which is lower than a study that estimated around 78.1% [11]. However, our result was greater than the 25.0% and 25.9% reported in studies done in Shandong Province [10, 24]. Differences in loneliness prevalence could be attributed to differences in the socio-economic development of the sampling age groups [10, 25]. This could also be explained by the different sample sizes and measuring tools used in the other studies. For instance, Zhang et al. [10] interviewed 5514 older residents who were also asked to assess their degree of loneliness via a single-item question in their study, while Wang et al. [11] surveyed and assessed 5652 rural older adults on their loneliness using the UCLA (the University of California at Los Angeles)-Loneliness Scale (20 items).

Our major findings in this study are that some aspects of functional health were associated with loneliness, while self-rated health was not. More specifically, we found that having limited functional ability and depression had a combined effect on loneliness, which is consistent with previous research [3, 26, 27], indicating that older people with limited or impaired functionality and depressive disorders may experience adverse psychological effects that included loneliness. Functional ability was the strongest predictor of loneliness in the current study, with depressive symptoms coming in second among all samples, highlighting the importance of maintaining good functional capabilities and mental health in later life [25]. Furthermore, marital status was found to be associated with loneliness, consistent with previous studies which reported that being widowed or divorced was accompanied by a higher risk of experiencing loneliness [5, 28]. Our results show that older people with functional inability, depressive symptoms, and being single (never married, divorced, or widowed) should be given special attention. Measures or programs to reduce loneliness should be targeted to this particular group.

Despite finding a relationship in the correlation statistic, no complex association of self-rated health in the regression tree model was observed. The independent contribution of self-rated health to loneliness has been reported in previous studies [9, 29]. However, it could have been attenuated with the presence of functional ability because previous studies reported that functional inability could cap social engagement and social connection, eventually resulting in loneliness [14].

The results showed that the complex associations of variables of loneliness partly differed between rural males and females. Variables common to both were functional ability and depressive symptoms, and this has also been supported by previous studies [3, 26, 27]. Our results revealed an association between marital status, functional ability, and depressive symptoms as variables of loneliness. On the other hand, marital status was found in older females but not in males, showing the variability in the complex associations between functional health and loneliness. This finding may be supported by a study which showed that males and females had different marital role expectations and social relationship structures in later life [30]. In addition, the findings of gender differences were not inclusive, implying that more attention should give to this issue. For example, most previous studies found that older women were more likely to suffer from loneliness and were more vulnerable to the negative health impact of loneliness [11, 25]. At the same time, there have also been reports suggesting that such differences do not exist [31]. However, the researchers found that older men were more likely to experience loneliness than women [11]. Further research is, therefore, necessary in this area.

Most studies that analyzed the correlations of loneliness all fitted linear regression models like logistic regression, multivariate regression, and hierarchical regression [3–8], which are helpful and valuable in finding direct predictors of loneliness. However, loneliness manifests itself in a more complex way in practice. For example, a Czech study found that loneliness was also significantly related to different educational backgrounds and residential areas, but in a complex non-linear way among older people [28]. Similarly, a Finnish cohort study found that living status and self-rated health had a combined effect on the onset of loneliness, suggesting that older people with poor self-rated health and who lived alone were more likely to experience loneliness [9]. Examining the complex associations or the combined effect of factors on the onset of loneliness could therefore allow us to better determine which subgroups are the most at risk of developing loneliness, eventually assisting in planning intervention programs to alleviate loneliness.

The advantage of our study using CART model may be that it computed multi-associations of analyzed variables to create a simple but robust regression tree that facilitated the investigation of the preliminary mechanism for developing loneliness. This is critical for developing fact-based results and focused programs to reduce loneliness among older adults in rural communities. Nevertheless, the current study cannot draw causal-relationship conclusions because of the cross-sectional study design data. The above findings should thus be interpreted with caution. Empirical studies focusing on exploring and developing the methods of putting these findings into practice should be encouraged. Besides, we only treat loneliness as a general concept and do not measure loneliness from an emotional and social perspective. In addition, we need to acknowledge that dichotomization might compromise the accuracy of the results. Also, the variables involved were not comprehensive, and other variables such as social isolation and solitude, which were distinct from loneliness, were not well considered.

## Conclusion

This paper identified the complex associations between depression, functional ability, and loneliness in China rural communities, discovering that older people with limited functional ability and depression are more likely to experience loneliness. Moreover, particular attention should be given to this subgroup and older single females to establish effective measures to reduce loneliness. Our results may provide accurate data for comprehensive interventions and help implement policies to reduce the incidence and development of loneliness among older people in rural communities.

## Data Availability

The datasets generated during and/or analyzed during the current study are available from the corresponding author on reasonable request.

## Abbreviations

ADL: Activities of daily living
IADL: Instrumental activities of daily living
SDS: Zung Self-Rating Depression Scale
CART: Classification and Regression Tree
